# Novel bone repairing scaffold consisting of bone morphogenetic Protein-2 and human Beta Defensin-3

**DOI:** 10.1186/s13036-021-00258-5

**Published:** 2021-02-08

**Authors:** Wei He, Daixu Wei, Jun Zhang, Xiaonan Huang, Da He, Bo Liu, Qilong Wang, Mingming Liu, Ling Liu, Yajun Liu, Wei Tian

**Affiliations:** 1 Department of Spine Surgery, Beijing JiShuiTan Hospital, 4th Medical College of Peking University, No.31 Xinjiekou East Street, Xicheng District, Beijing, 100035 China; 2 Department of Biomaterials and Microorganisms, Northwest University, Xi’an, China; 3 Department of Spine Surgery, Zhejiang Provincial People’s Hospital, Hangzhou Medical College People’s Hospital, Hangzhou, Zhejiang China; 4 Department of Chemistry, Capital Normal University, Beijing, China; 5 Department of Gynaecology and Obstetrics, Third Medical Center of Chinese PLA General Hospital, Beijing, China

**Keywords:** Artificial bone scaffold, Bone repair, Phospholipid modified protein, Bone injury

## Abstract

**Background:**

Synthetic biomaterials assist in modulating the vascular response in an injured bone by serving as delivery vehicles of pro-angiogenic molecules to the site of injury or by serving as mimetic platforms which offer support to cell growth and proliferation.

**Methods:**

This study applied natural phospholipid modified protein technologies together with low temperature three-dimensional printing technology to develop a new model of three-dimensional artificial bone scaffold for potential use in repairing body injuries. The focus was to create a porous structure (PS) scaffold of two components, Bone Morphogenetic Protein-2 and Human Beta Defensin-3 (BMP2 and hBD3), which can synchronously realize directional bone induction, angiogenesis and postoperative antibacterial effects. BMP2 induces osteogenesis, whereas hBD3 is antibacterial.

**Results:**

Our data showed that in the BMP2-hBD3-PS or hBD3-PS scaffolds, BMP2 had a slow-release rate of about 40% in 30 days, ensuring that BMP2 could penetrate into stem cells for osteogenic differentiation for a long time. The scaffolds promoted cell growth when in combination with BMP2, thus showing its importance in promoting cell growth. Alkaline Phosphatase (ALP) staining showed that the ALP content of BMP2-hBD3-PS and BMP2-PS had a significant increase in samples that contained BMP2, thus showing that these scaffolds promoted osteogenic differentiation. In all the constructs that had hBD3, they displayed antibacterial properties with hBD3, having a slow release of about 35% in 30 days, thus ensuring they provided protection.

**Conclusion:**

Based on this study, the 3D printed BMP2 scaffolds show a great potential for the development of biodegradable bone implants.

**Level of evidence:**

Level II, experimental comparative design.

## Background

Annually, over 15 million people around the globe suffer from bone injuries as a result of accidents or diseases, with approximately 10% being secondary to complications of bone non-union as a result of unsuccessful repair or recovery [1, 2]. The autogenous bone graft is the gold standard of bone repair due to its osteoinductivity, osteoconductivity, and osteogenecity [3] and this involves the harvesting of an osseous graft from a single anatomic site and transplanting to another part within the same individual. It has the advantages of rapid and complete integration into the host bone site. The main disadvantages are that it is associated with higher risks of donor site morbidity, postoperative pain, increase in blood loss, and infections [4, 5].

Older people are more prone to vertebral column injuries, with typically prolonged healing periods. At the spinal cord injury (SCI) site, the microenvironment is complicated, and thus there is need of more than one process to be regulated for axonal regrowth to occur. Minimization of aggravating factors such as gliosis or inflammation is of utmost importance in order to accelerate regeneration of injured axons [6, 7]. Although axons of injured spinal cords possess a potential to regenerate various pathophysiological changes, the complications that accompany the injury hinder such regeneration. In order to overcome this hurdle, neural tissue engineering has been considered as a probable option with the creation of biomaterial scaffold being synthesized from natural or synthetic polymer. It can help prevent scar tissue formation and concentrate neurotrophic growth factors while promoting axonal regeneration between the two ends of the injured neural tissue [8, 9].

While fabricating implantable scaffolds to treat SCI, various parameters need to be considered and these include: biocompatibility, biodegradability, mechanical strength, scaffold morphology and internal matrices [9]. Biomaterials assist in modulating the vascular response following SCI through two distinct mechanisms: by acting as vehicles for the delivery of pro-angiogenic molecules [10] or by serving as extracellular matrix (ECM) mimetic platforms which offer support to cell growth and proliferation [11]. Biomaterials have the ability to protect cells and therapeutic agents from the harsh conditions found in SCI scar site and are key to the development of targeted regenerative therapies. This is complemented by the biocompatible and biodegradable characteristics [12]. Biocompatibility is important as it decreases the risk of triggering toxic or immunological responses within the central nervous system and prevents the induction of chronic inflammation [13, 14].

3D printing technology has been successfully used in the preparation of scaffolds with adjustable morphologies. It has however been recognized that this method has its limitation in the hydrophobic surfaces, limited osteo-inductive ability and lack of bioactivity, thus limiting their application in bone repair [15]. Various strategies have been put in place to address this shortcoming by performing a surface modification incorporating the bioactive factor into the 3D scaffolds in order to accelerate bone healing. Among osteogenic-related bioactive factors, the bone morphogenetic protein-2 (BMP2) which is a relevant factor in bone defect repair, has been generally used in tissue engineering approaches for the repair of bone injuries and defects. BMP2 can improve gene expression during osteogenic differentiation in vitro, including that of osteopontin, osteocalcin, bone sialoprotein and alkaline phosphatase (ALP) [16, 17].

Human mesenchymal or skeletal stem cells (SSC), derived from the bone marrow when seeded onto nanofibrous scaffolds, have the ability to differentiate into an osteogenic lineage [18]. A combination of osteogenic growth factor release (BMP2) from polymer scaffolds improves bone regeneration during bone engineering.

Both growth factors and antimicrobial cationic peptides are typical hydrophilic proteins, while the vast majority of polymer materials are hydrophobic, so it is mainly reported that active factors are grafted on the polymer surface by chemical reaction or physically adsorbed. Most reports use other hydrophilic biomaterials attached to the surface to add active factors into polymer scaffolds. However, the scaffolds [19, 20] prepared by these traditional techniques only contain a small level of active factors, and the loaded proteins are easily inactivated.

In recent years, some researchers apply liposome principle for reference, and try to use nontoxic and harmless amphiphilic biosurfactant phospholipid to self-assemble on protein surface (phospholipid-modified protein). The hydrophilic end of phospholipid faces hydrophilic protein, while the hydrophobic end faces organic solvent, forming a phospholipid protective layer, and improving the dispersion of protein in organic solution, so as to achieve the purpose of sustained release of protein [21]. Concomitantly, phospholipids play an important role in protecting proteins, avoiding the degradation and destruction of chloroform and protease in the external environment.

The protective effect of phospholipid technology on protein factors has been affirmed, but at present, there is no report on the combination of phospholipid technology and polymer materials. Therefore, exploring whether it can protect and release protein factors in polymer materials will have great influence on the significance of bone tissue engineering.

In recent years, foreign research teams have explored a new low-temperature 3D printing technology (LTP), which is gradually applied in the field of medical tissue engineering [22, 23]. Comparing with traditional three-dimensional printing technology with high temperature melting method as printing condition, LTP technology works at − 20 °C ~ 4 °C, which can effectively avoid the interference of high temperature environment on protein activity [23, 24]. Therefore, low temperature three-dimensional printing technology promises to be a reliable method to prepare tissue engineering scaffolds loaded with active factors in the future.

This study aimed to apply the latest natural phospholipid modified protein technologies together with low temperature three-dimensional printing technology to develop a new model of three-dimensional artificial bone scaffold and explore its mechanism of repairing vertebral body injuries.

## Methods

### Preparation of scaffolds

The preparation method proposed by Wei et al. [25, 26] was followed with slight modification. Briefly, BMP2 and human Beta Defensin 3 (hBD3) were mixed in Phytohaemagglutinin (PHA). Scanning electron microscope (SEM) observations of three-dimensional scaffold were done before printing multifunctional support at low temperature by a 3D printer.

The micro-morphology of the samples was observed by SEM. The samples were placed in a vacuum dryer for approximately 1 h at room temperature, cut into 10–15 mm lengths and fixed on the copper plate with double-sided conductive tape specially used for SEM. After gold spraying, the surface of the three-dimensional printing multifunctional bracket was observed and the voltage was 15 kV during observation.

### Cell culturing

Human adipose-derived mesenchymal stem cells (hADSC) were selected for research and were purchased from the cell bank of Chinese Academy of Sciences.

Standardized cell culturing techniques were implemented, using Dulbecco’s modified Eagle’s medium (DMEM) as cell culture medium.

### Biocompatibility test of three-dimensional printing multifunctional bracket

CCK-8 method was used to test the biocompatibility of 3D printing multifunctional stent. The prepared 3D printing multifunctional scaffold was cut into a size similar to that of a 48-hole plate and soaked in 75 wt% ethanol solution for 12 h for disinfection. It was then soaked in Phosphate-buffered saline (PBS) and Dulbecco’s modified Eagle’s medium (DMEM) for 2 h to remove residual ethanol, then placed in a 48-well plate. Each well was planted with 1 × 10^5^ cells, which were cultured for 1, 4 or 7 days, respectively. Every two days, the fresh DMEM medium was changed. When the specific culture time was reached, the culture solution was discarded, and 300 μL of fresh DMEM containing 10% (v/v) CCK-8 was added to continue the culture for two hours. Consequently, 100 μL of the reaction solution from the 48-well plate was added to a new 96-well plate, and OD reading was carried out by using a microplate reader at the wavelength of 450 nm. Six parallel samples (*n* = 6) were taken from each sample.

### Confocal laser scanning microscopy observation of cell growth in three-dimensional printing multifunctional scaffold

The preliminary operation is similar to the biocompatibility test of a 3D printing multifunctional stent. The prepared 3D printing multifunctional scaffold was cut into a size similar to that of a 48-hole plate and soaked in 75 wt% ethanol solution for 12 h for disinfection. It was then soaked in PBS and DMEM for 2 h to remove the residual ethanol and afterwards placed in a 48-well plate. Each well was planted with 1 × 105 cells, which were cultured for 1 day, 4 days and 7 days in turn.

Every two days, the fresh DMEM culture medium was changed. When the culture reached the specific time point, the culture solution was removed. To clean the 3D printing multifunctional scaffold, PBS was used to remove the residual DMEM. Fixation was done with 4%(v/v) paraformaldehyde at room temperature for 30 min, and the 3D printing multifunctional scaffold was washed repeatedly with PBS three times so as to remove the residual paraformaldehyde. Phalloidine-Alexa Fluor® 488 (green) was then added and after incubation, this solution was discarded. The three-dimensional printing multifunctional stent was washed with PBS three times repeatedly to remove the residual phalloidin e-Alexa fluor 488 solution. The number and community morphology of three-dimensional printing multifunctional scaffold cells were observed under the excitation wavelength of 488 nm by a laser confocal microscope. Three parallel samples (*n* = 3) were taken from each sample.

### Quantitative detection of ALP in osteogenic differentiation

Osteogenic medium was prepared that consisted of complete culture media, such as an α-Minimum essential medium (α-MEM), nonessential amino acids supplemented with 10% fetal bovine serum (Gibco, Life Technologies), 100 U/mL penicillin, 0.2 mg/mL streptomycin; supplemented with 100 nM dexamethasone, 50 μM ascorbic acid and 10 mM β–glycerophosphate (Sigma-Aldrich, St. Louis, MO, USA). Washing buffer was made of Calcium-magnesium free PBS with 0.05% tween 20, BCIP/NBT Tablet dissolved in 10 mL of distilled water. Fixation of cells was done in 10% formalin.

96 well-plates were coated with 200 μL of varied concentrations of the scaffold in culture media. The plates were then centrifuged at 2000 rpm for 2 min at room temperature. 100 μL media was removed and 3 X 103/100 μL hADSC were seeded on them with and without differentiation media. Incubation was done for 5 and 15 days accordingly. The cultured cells were then washed with PBS twice and cellular membranes were lysed by 200 μL lysis buffer (Tris-HCl 25 mM, TritonX-100 0.5%) at 4 °C for 2 h. Following completion of lysis, 50 μL lysate was aliquoted into another 96 well plate and 50 mmol/L p-nitrophenylphosphate (p-NPP) in a sodium carbonate buffer at pH 10.4 was added to cell lysate, followed by incubation 37 °C for 30 min in an incubator. The amount of released p-nitrophenylphosphate was estimated by measuring the absorbance at 405 nm by spectrophotometric method. The quantity of p-nitrophenol (pNP) in each well was determined using a standard curve established using p-NPP and purified ALP enzyme.

### Antibacterial detection

The microorganisms used were *E. coli* and *S. aureus* obtained from laboratory stock. These microbes were grown to mid-logarithmic phase in tryptic soy broth and then diluted to 106 CFU/mL in 10 mM potassium phosphate 1% tryptic soy broth (pH 7.4). 100-μL portions of cells were incubated in the presence of different concentrations of scaffolds for 3 h at 37 °C. Serial dilution of the cells was then performed. The cells were plated on and bacterial viability determined after 1 h and 24 h.

## Results

**Fig. 1:**
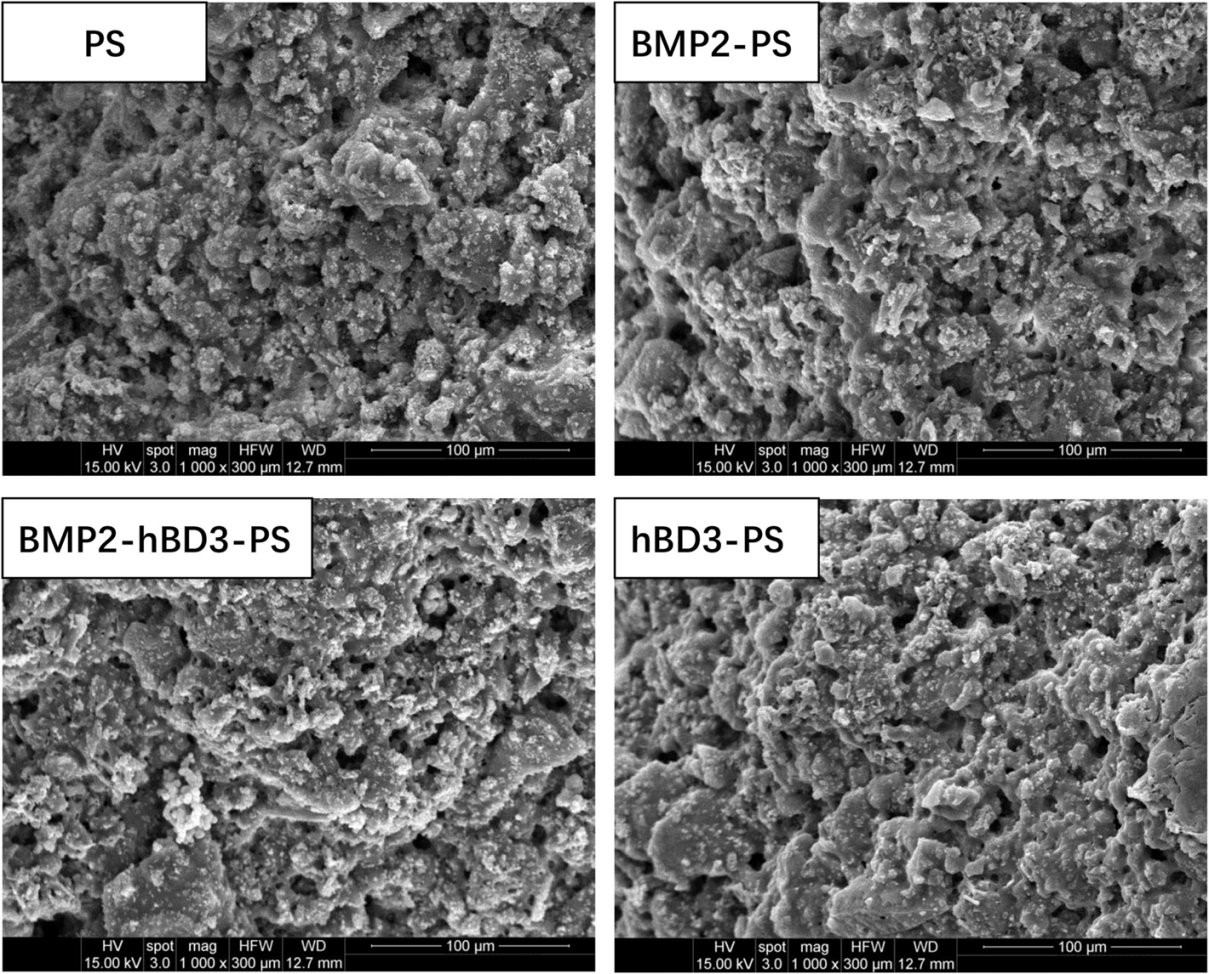
Microstructure of all samples. The results show that the microstructure of all samples is similar, the main body is PHA material, and the micro surface contains a small amount of porous structure (PS)

The results show that the microstructure of all samples is similar, the main body is PHA material, and the micro surface contains a small amount of porous structure (PS) as shown in Fig. 1.

### Determination of sustained release curves of BMP2 and hBD3

**Fig. 2:**
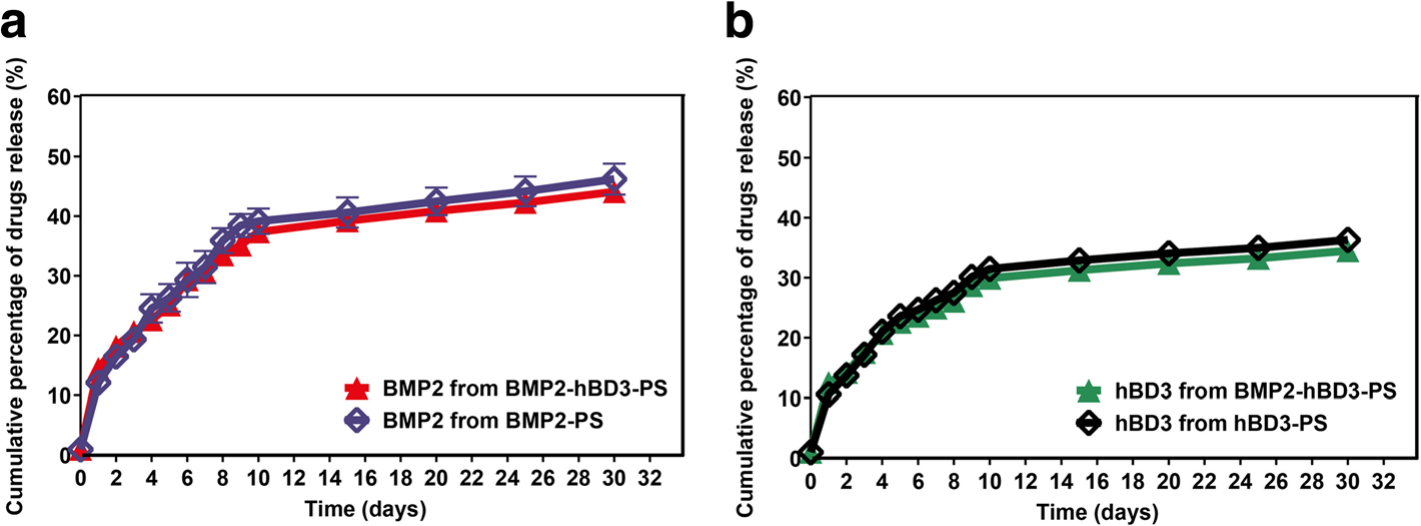
**a** BMP2 release curves BMP2-hBD3-PS and BM2-PS; **b** Release curves of hBD3 from BMP2-hBD3-PS and BM2-PS. For both BMP2-hBD3-PS and BMP2-PS, the slow-release curve of BMP2 was close. Furthermore, the release rate was only about 40% in 30 days. Regardless of BMP2-hBD3-PS or hBD3-PS, the slow-release curve of hBD3 is close as well with a release rate in 30 days of roughly 35%

The results showed that for both BMP2-hBD3-PS and BMP2-PS, the slow-release curve of BMP2 was close. Furthermore, the release rate was only about 40% in 30 days, which ensured that BMP2 could penetrate into stem cells for osteogenic differentiation for a long time. Regardless of BMP2-hBD3-PS or hBD3-PS, the slow-release curve of hBD3 is close as well with a release rate in 30 days of roughly 35% (Fig. 2) which is considerably slow-release and ensures that hBD3 can resist bacteria for a long time.

**Fig. 3:**
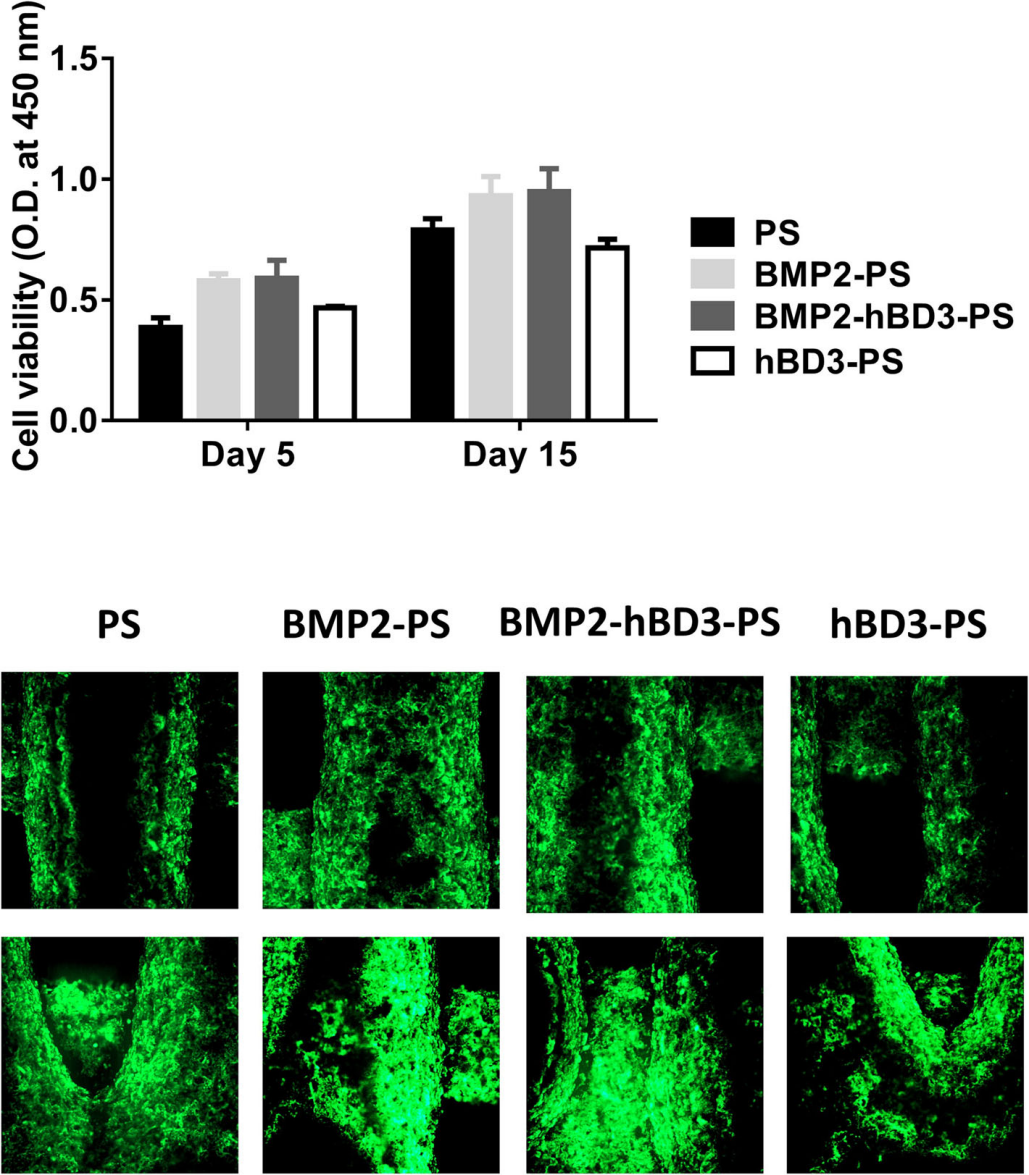
Cell proliferation for different scaffolds. All scaffolds promote the cell growth, though PS and hBD3-PS without BMP2 had poor cell growth ability. It shows that BMP2 can promote cell growth to a certain extent

As shown in Fig. 3, all scaffolds would promote the growth of cells, but PS and hBD3-PS without BMP2 had poor cell growth ability. It shows that BMP2 can promote cell growth to a certain extent.

**Fig. 4:**
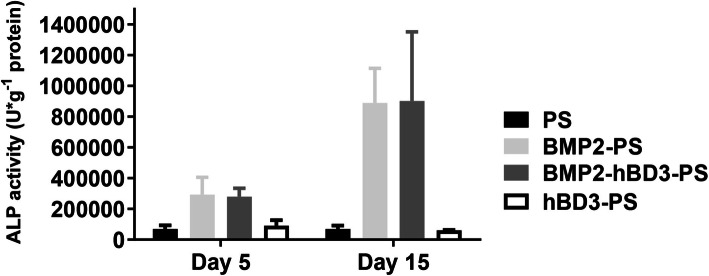
ALP activity results. The ALP content of BMP2-hBD3-PS and BMP2-PS increased significantly in samples containing BMP2, while the ALP content of PS and hBD3-PS in samples without BMP2 was insignificant and extremely low

The ALP content of BMP2-hBD3-PS and BMP2-PS increased significantly in samples containing BMP2, while the ALP content of PS and hBD3-PS in samples without BMP2 was insignificant and extremely low (Fig. 4). The results indicate that the scaffold containing BMP2 can effectively promote the osteogenic differentiation of ADSC without major differences.

### Antibacterial activity

**Fig. 5:**
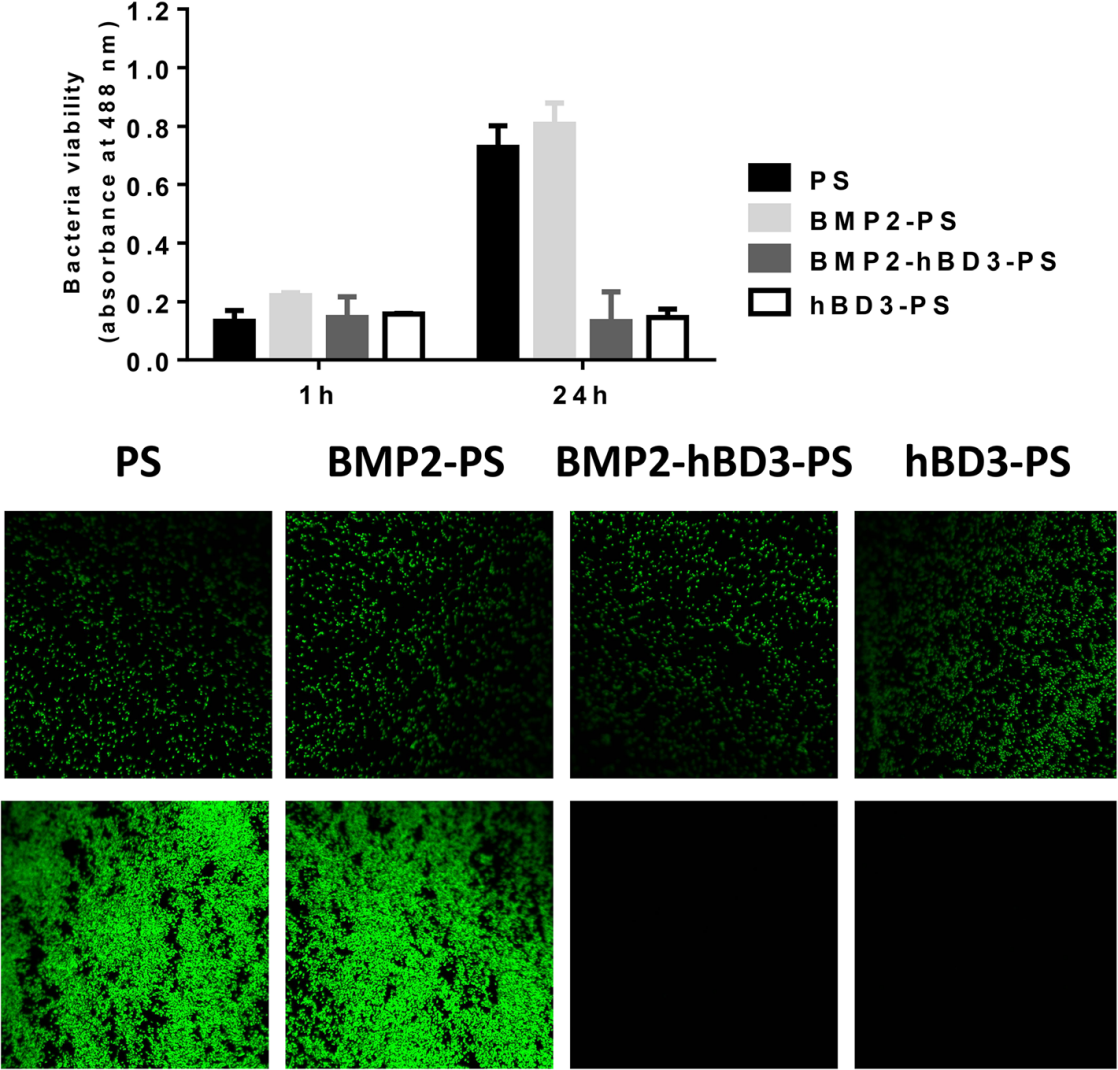
Antibacterial activity results. Unlike the overall growth trend of cells, BMP2-hBD3-PS and hBD3-PS had significant antibacterial ability in samples containing hBD3. Compared with samples without hBD3, PS and BMP2-PS had almost no antibacterial ability, and the bacteria grew significantly

Unlike the overall growth trend of cells, BMP2-hBD3-PS and hBD3-PS had significant antibacterial ability in samples containing hBD3. Compared with samples without hBD3, PS and BMP2-PS had almost no antibacterial ability, and the bacteria grew significantly (Fig. 5). Therefore, the scaffold containing hBD3 has significant long-term antibacterial ability and does not affect the ability of BMP2 to promote stem cell differentiation.

## Discussion

SCI has serious consequences as it affects the quality of life, life expectancy, and causes a serious economic burden as its treatment is associated with high costs and a patient’s loss of income. More often, SCI results in paralysis as well as reduced cardiovascular, gastrointestinal, urinary, and sexual functions [27]. Much effort has been directed towards reducing secondary injury and enhancing tissue sparing. However, pathway repair after complete transections or after chronic injury need implantation of some focused bridging structures with the goal to restore the tissue continuity in the area of trauma. The scaffolds need to be biocompatible to create an environment that promotes tissue growth and vascularization and enhances the regeneration of axons [28].

The modification of BMP2 to be spread in a polymer solution and keep its function is key of the loading and release of bioactive BMP2 for polymer substrates. In our study, soybean phospholipid (SL), which is a common amphiphilic substance in cells, was selected to modify two active factors: BMP2 and hBD3. The results showed that the slow-release curve of BMP2 was similar, with a release rate of about 40% in 30 days, ensuring that BMP2 could penetrate into stem cells for osteogenic differentiation for a long time, irrespective of whether BMP2-hBD3-PS or hBD3-PS was used. This outcome of the experiment compares well with that of prior publications. In a study by Qu et al. they demonstrated that poly lactic-co-glycolic acid (PLGA) film loaded with over 80 wt% BMP2, which was regarded as substrate-promoting osteoblast attachment, proliferation and differentiation for application of bone tissue engineering was superior. Based on phospholipid as a surfactant, BMP2 was modified as a complex (PBC) for dispersing in PLGA/dichloromethane solution [29].

In order to test bone induction and angiogenesis, human adipose-derived mesenchymal stem cells (hAMSC) were utilized. The findings of this study showed that all the scaffolds were capable of promoting growth of cells. Nevertheless, the PS and hBD3-PS without BMP2 had poor cell growth ability thus showing that BMP2 is vital for the promotion of cell proliferation. This is supported by observations in previous studies that have shown BMP2 as important in promotion of cell growth leading to its utilization with different scaffolds that aim to ensure its long-term delivery due to its short half-life. Using mouse mesodermal progenitor cells, Wang et al. demonstrated that high concentrations of BMP2 induced their differentiation into chondrocytes and bone cells [30]. In other studies, BMP2 has been implicated in the conversion of rat calvaria derived multipotent cells and clonal myoblast cells into cells of the osteoblast phenotype [31]. A study by Kanatani et al. demonstrated that BMP2 stimulated bone resorption as a result of stimulating osteoclast formation and activating mature osteoclasts stromal cells of mouse bone cell cultures [32].

To determine if early osteogenic differentiation of cells was induced by BMP2, ALP staining was performed. The results illustrated that ALP content of BMP2-hBD3-PS and BMP2-PS had a significant increase in samples that contained BMP2, whereas the ALP content of PS and hBD3-PS in samples that were without BMP2 had no significant change. These results show that the scaffolds containing BMP2 were capable of promoting osteogenic differentiation of ADSC. These results are similar to those of a study by Jian et al. which demonstrated that BMP2 facilitated the osteogenic differentiation of bone marrow stem cells through the induction of ALP activity, thereby promoting mineralization, enhancing adherence and mediating the expression and activation of certain associated osteogenic markers [33].

Lack of antibacterial properties presents a serious challenge to the biomedical to the application of biopolymers such as BMP2. Several studies have shown that antibacterial components can be incorporated into the scaffold construct evoking immunological responses. In the current study, BMP2-hBD3-PS and hBD3-PS had significant antibacterial ability in samples containing hBD3. In comparison to samples without hBD3, PS and BMP2-PS had almost no antibacterial ability. The bacteria grew significantly showing that the antibacterial activity in the scaffold constructs was attributable to hBD3 which had a significant antibacterial effect and did not affect the ability of BMP2 to promote stem cell differentiation. It has been demonstrated that hBD3 exhibits antibacterial activities against both Gram-negative and Gram-positive bacteria and equally has the ability to act as a chemo-attractant [34]. Therefore, this study demonstrated the efficacy of hBD3 in preventing bacterial infections during bone healing without affecting the activity of BMP2. HBD3 had a slow release of about 35% in 30 days which ensures that it can provide an appropriate long-term bacterial resistance.

## Data Availability

The raw data used and/or analyzed during the current study are available from the corresponding author on reasonable request.
